# Monocyte Single‐Cell‐Type Gene Expression Analysis Differentiates Kawasaki Disease From Viral Infection With High Specificity

**DOI:** 10.1111/jcmm.71303

**Published:** 2026-07-29

**Authors:** Nelson L. S. Tang, Tsz‐Ki Kwan, Assunta C. H. Ho, Hugh Simon Lam, Geoffrey Mok, Dan Huang, Suk‐Ling Ma, Kwong‐Sak Leung

**Affiliations:** ^1^ Department of Chemical Pathology and Li Ka Shing Institute of Health Science, Faculty of Medicine The Chinese University of Hong Kong Hong Kong China; ^2^ Cytomics Limited Hong Kong Science Park Hong Kong China; ^3^ Hong Kong Branch of CAS Center for Excellence in Animal Evolution and Genetics and KIZ/CUHK Joint Laboratory of Bioresources and Molecular Research in Common Diseases Hong Kong China; ^4^ Functional Genomics and Biostatistical Computing Laboratory CUHK Shenzhen Research Institute Shenzhen China; ^5^ Department of Paediatrics, Faculty of Medicine The Chinese University of Hong Kong Hong Kong China; ^6^ Southern University of Science and Technology Shenzhen China; ^7^ School of Arts and Humanities Tung Wah College Hong Kong China; ^8^ Department of Computer Science and Engineering, Faculty of Engineering The Chinese University of Hong Kong Hong Kong China

**Keywords:** biomarker, DIRECT LS‐TA, interferon, Kawasaki disease, single‐cell‐type gene expression, viral infection

## Abstract

Kawasaki disease (KD) and viral infection (VD) share similar clinical features but require distinct treatments. A practical biomarker to distinguish them is therefore clinically important. Previous blood transcriptome studies identified many differentially expressed genes, but large gene panels are impractical for routine laboratories. A ratio‐based Direct Leukocyte Single‐cell‐type Transcript Abundance (DIRECT LS‐TA) assay was recently developed to quantify monocyte gene expression directly in whole blood using a monocyte‐specific target gene relative to monocyte reference genes (*PSAP* or *CTSS*). Interferon‐stimulated genes *IFI27, IFI44L*, and *SIGLEC1* can be measured by this approach. In this study, three ratio biomarkers (*IFI27/PSAP, IFI44L/PSAP, SIGLEC1/PSAP*) and a conventional interferon (IFN) score derived from eight genes were calculated from public blood transcriptome datasets (GSE73461 and GSE68004) and compared between KD and VD. VD patients showed markedly elevated IFN‐related biomarkers, with all three ratios significantly higher in VD and *IFI27/PSAP* giving the largest increase. *IFI27/PSAP* achieved the highest diagnostic performance (AUC 0.90), slightly exceeding the conventional IFN score (AUC 0.89). These findings suggest that absent or minimal IFN activation argues against VD and supports KD, and that this simple ratio assay could serve as a clinically useful exclusion test.

**Abbreviations**
CRP: C‐reactive protein
DEG: Differentially expressed gene
DIRECT LS‐TA: Direct Leukocyte Single‐cell‐type transcript abundance
IFN: Interferon
ISG: Interferon‐stimulated gene
IVD: In vitro diagnostics
KD: Kawasaki disease
PB: Peripheral blood
RBB: Ratio‐based biomarker
VD: Viral infection
WB: Whole blood

## 1 Introduction

In paediatric patients, differentiating between Kawasaki Disease (KD) and viral infection (VD) is a clinical challenge due to overlapping symptoms [1, 2]. Febrile illness is a common clinical presentation in children. While blood culture and molecular methods can identify specific viral pathogens, these approaches can be time‐consuming and do not always identify the causative agent. KD requires prompt administration of intravenous immunoglobulin to prevent coronary artery complications, while viral infections typically require only supportive care [3].

Current diagnostic approaches often rely on the detection of specific pathogens by culture or molecular methods, which can be time‐consuming [4, 5]. While the host responds differently to different categories of pathogens, biomarkers targeting host response are less well developed. Currently, only a few host response biomarkers are available, and most of them are serum proteins, such as C‐reactive protein (CRP) and procalcitonin. They can indicate the presence of inflammation; however, they lack the specificity required to differentiate KD from VD.

Differentiation of KD and VD is an important clinical decision as their treatment is completely different. Making the right diagnosis early in the course of illness plays a paramount role in a good outcome. Although various high‐technology biomarkers (like transcriptomic biomarkers, microarray or RNA sequencing) have been explored with good differentiation, the potential for routine application of these costly biomarkers in routine health care or developing countries is limited [6]. Therefore, a simple biomarker that can be measured by widely accessible machines, like qPCR machines, is more desirable.

Viral infection leads to activation of the interferon (IFN) pathway, particularly Type I interferon, in epithelial cells infected by the virus. Interferon‐stimulated genes (ISGs) play a fundamental role in the innate immune system and act as inhibitors of viral replication in infected cells and have a defensive action in uninfected cells [7, 8]. The levels of expression of a battery of interferon‐stimulated genes (ISGs) in peripheral blood (commonly known as interferon score) have been first developed as a bioassay of systemic IFN responses in autoimmune diseases [9].

With the advances in molecular techniques to quantify gene expression, many researchers analysed peripheral blood of KD patients by microarray or RNA‐sequencing [10, 11, 12, 13, 14, 15, 16]. Using the method called differential expression gene (DEG), the expression of each gene is statistically analysed one by one, and then the genes with the greatest expression difference between different groups were identified as the biomarker. However, as shown in Table 1, previous blood transcriptomic studies in KD reported almost completely different sets of DEGs. It is uncertain which study is correct or even if KD occurs due to a single distinct inflammatory mechanism. An important reason for such discrepancies in DEG gene panels found among KD studies is that all bulk sample analysis methods ignore the confounding factor of the cell counts of various cell subpopulations and their changes in different diseases. Therefore, variations in cell count confounded the output of DEGs heavily and may explain why DEGs found in different studies were largely different. Therefore, a gene expression analysis method that is independent of cell count proportion is needed, and thus recently many researchers used single‐cell RNA sequencing to study gene expression changes in various leukocyte cell types in PB. However, single‐cell RNA sequencing is too expensive and cannot be afforded by many patients.

**TABLE 1:** Previously reported DEGs on KD.

Published studies	Reported genes	References
Kuiper et al., 2023, Wright et al., 2018	*CACNA1E, KLHL2, PYROXD2, SMOX, ZNF185, LINC02035, CLIC3, S100P, IFI27, TIGIT, CD163, RTN1*	[14, 17]
Liu et al., 2021	*IL1B, ADM, PDGFC, TGFA*	[11]
Nie et al., 2021	*CXCL8, CCL5, CCR7, CXCR3, CCR1*	[12]
Hoang et al., 2014	*MMP‐8, CEACAM1, PFKB2, ASPRV1, CYP26B1, TRANK1, NKX3‐1, IL1B, IL1R1, IL1R2, IL1RAP, IL1RN*	[18]
Popper et al., 2009	*MX1, MX2, ISG15 (G1P2), IFIT2, OAS1, OAS2, PARC, ITGA2B, ITGB5, TUBB1, TBCC, BPI, SLPI, DEFA1, PI3, S100P, S100A12, MMP9, SORL1, PBEF, IL‐1R1, KBKG, PIK3R1, PRKAR1A, PAX5, CD79A, MS4A2, IGHM, SPIB, HLA‐DNA, TRAF5*	[4]

To address these limitations, the Direct Leukocyte Single‐Cell‐type Transcript Abundance (DIRECT LS‐TA) method has emerged as a promising approach for quantifying cell‐type‐specific gene expression directly from bulk blood samples. This method leverages the knowledge of cell‐type proportions and differential gene expression profiles to identify monocyte‐informative genes. By focusing on genes that are predominantly expressed in specific leukocyte subpopulations, DIRECT LS‐TA minimises the confounding effects of cell count variations and provides a more refined assessment of the host response. Single‐cell‐type (monocyte) informative genes are shortlisted genes whose transcripts account for more than 50% of transcripts in the bulk (cell mixture) samples, such as peripheral blood (PB). These genes could be visualized in iceberg plots, which are specially designed for direct LS‐TA applications. The extent of higher expression of genes in purified monocytes over peripheral blood (cell‐mixture sample) is shown on the y‐axis on iceberg plots. Those genes, such as *IFI27*, that are above the water level, which is the threshold of 2.5‐fold on the y‐axis, indicates that their RNA transcripts in the bulk peripheral blood samples predominantly originate from monocytes [19, 20, 21].

The hyperinflammatory immune dysfunction in KD is characterised by activation of the TNF and IL‐1 signalling pathways [22]. They are also implicated in the initiation of pathology of the cardiovascular system. High levels of TNF‐alpha and IL‐1 beta and their downstream signals were reported in the pathogenesis and disease development of KD [23, 24]. TNF‐alpha further enhances the production of proteases like matrix metalloproteases (MMPs), which degrade extracellular matrix and cause vascular damage [24, 25]. These findings confirmed the role of these pro‐inflammatory cytokines in KD. On the other hand, type I interferons (as a distinct axis of the host response) do not play an important role in KD.

The ratio‐based biomarker (RBB) approach is the second pillar of the DIRECT LS‐TA; it enables the use of 2 ‘above‐the‐waterline’ monocyte genes to reveal target genes (e.g., *IFI27*) as the numerator and a stably expressed gene (e.g., *PSAP*) as the denominator to represent single‐cell‐type‐specific gene expression from bulk blood samples without the need for complex cell separation techniques. We recently showed that monocyte‐specific RBBs (*IFI27/PSAP*) had superior performance in the detection of viral infection [21]. A simple ratio‐based biomarker is more ready for implementation in hospital laboratories as it is much easier to standardise and no special machine is required for multi‐gene quantification. Here, we evaluate three monocyte‐specific RBBs (*IFI27/PSAP*, *IFI44L/PSAP*, and *SIGLEC1/PSAP*), which are also ISGs, for differentiating KD from VD, aiming to develop a practical and efficient diagnostic tool for clinical implementation.

## 2 Materials and Methods

### 2.1 Datasets Used in the Analysis

Gene expression datasets obtained from peripheral blood samples were collected from the Gene Expression Omnibus (GEO), maintained by the US National Institutes of Health. Two WB datasets (GSE73461 and GSE68004) containing both KD and VD patients were analysed (Table 2).

**TABLE 2:** List of WB gene expression datasets used for comparison of DIRECT LS‐TA and IFN score in differentiation of KD.

Dataset accession number	Grouping of samples and number of samples	Pathogen(s) included in the viral infection group	Type of blood sample	References
GSE73461 (Illumina microarray)	Viral infection group: 94 Kawasaki disease group: 77	RSV, adenovirus, parainfluenza 1–4, influenza A + B, bocavirus, metapneumovirus, rhinovirus/enterovirus	WB	[14]
GSE68004 (Illumina microarray)	Viral infection group: 18 Kawasaki disease group: 76	Adenovirus	WB	[26]

### 2.2 Development of DIRECT LS‐TA Value From Gene Expression Datasets

We showed how to select numerator and denominator genes for deriving an RBB representative of the expression of the numerator (target) gene in monocytes in PB samples [19]. To develop the ratio‐based biomarker (RBB), *PSAP* was selected as the denominator gene among the shortlisted monocyte informative genes. The denominator gene (monocyte informative reference gene) was selected based on having the least biological variation. Therefore, the percentage coefficient of variation (CV%) was calculated for each monocyte informative gene to find those with the lowest CV%. Conventional housekeeping genes were not useful in this approach as they are expressed across all cell types in peripheral blood but not specific to monocytes.

### 2.3 Calculation of DIRECT LS‐TA Parameters

The DIRECT LS‐TA was calculated as follows:

$$\text{Monocyte DIRECT}\;\mathrm{LS}-\mathrm{TA}=\frac{\text{monocyte informative gene}\left(\mathrm{WB}\right)}{\text{PSAP}\left(\mathrm{WB}\right)}$$

After log transformation:

$$\mathrm{Log}\;\left(\text{monocyte DIRECT}\;\mathrm{LS}-\mathrm{TA}\right)=\frac{\mathrm{Log}\;\left(\text{monocyte informative gene}\left(\mathrm{WB}\right)\right)}{\mathrm{Log}\;\left(\text{PSAP}\left(\mathrm{WB}\right)\right)}$$

$$\;=\mathrm{Log}\;\left(\text{monocyte informative gene}\;\left(\mathrm{WB}\right)\right)–\mathrm{Log}\;\left(\text{PSAP}\left(\mathrm{WB}\right)\right)$$

To convert to fold change against a healthy reference individual, multiples of the median (MoM) of monocyte DIRECT LS‐TA were used by subtracting monocyte DIRECT LS‐TA results of patients from the median in the control group. By setting the median of log Monocyte DIRECT LS‐TA of the control (KD) group to zero, a MoM of log DIRECT LS‐TA of each sample is similar to the delta–delta CT values in qPCR or dPCR experiments.

For comparison, we calculated the conventional interferon (IFN) score using the following formula:

$$\begin{array}{c} \mathrm{IFN}\;\text{score}=\text{average of}\;\log\;\left(\text{IFIT1},\text{IFITM3},\text{SIGLEC1},\mathrm{IFI}27,\mathrm{IFI}4\text{4L}\right)\\ \;-\text{average of}\;\log\;\left(\text{GAPDH},\text{ACTB},\mathrm{RPL}31\right) \end{array}$$

### 2.4 Statistical Analysis

Statistical analyses were performed using the R statistical package (R 4.2.1). Correlation analysis between DIRECT LS‐TA and continuous clinical variables was performed by Spearman correlation. Associations between DIRECT LS‐TA and categorical clinical variables were tested by the Wilcoxon Rank sum test (two categories). ROC curves were used to evaluate sensitivity and specificity for every possible cut‐off for the potential biomarkers in bulk RNA‐seq data. In general, *p* values < 0.05 were considered significant.

### 2.5 Evaluation of Biomarker Performance

Three RBB DIRECT LS‐TA biomarkers (*IFI44L/PSAP, SIGLEC1/PSAP, and IFI27/PSAP*) were evaluated for their ability to differentiate KD from VD. Area under the receiver operating characteristic curve (AUC‐ROC) analysis was performed to assess diagnostic performance. Additionally, sensitivity and specificity at various cutoff points were calculated to determine optimal diagnostic thresholds.

## 3 Results

Initial validation studies demonstrated that DIRECT LS‐TA values obtained from whole blood strongly correlated with gene expression measured in purified monocytes [19]. The correlation was particularly strong for *IFI44L/PSAP* (R^2^ ≥ 0.9), confirming that this ratio‐based biomarker effectively captures monocyte‐specific gene expression without the need for cell separation.

### 3.1 Differential Expression Analysis in VD vs. KD

**FIGURE 1:**
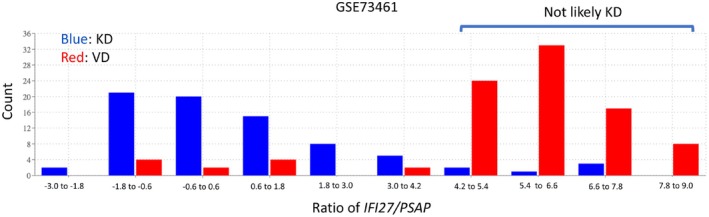
Distribution of values of ratio of *IFI27/PSAP* among KD and VD patients. *IFI27/PSAP* can be used as a screening tool for KD patients. Patients who are above the cut‐off value are unlikely to have KD.

Patients with VD had significantly higher expression of IFN biomarkers. All 3 RBB were significantly increased in VD (*p* < 1e‐5). *IFI27/PSAP* showed the largest increase in the VD group (Table 3). The distribution of *IFI27/PSAP* values was shown in Figure 1.

**TABLE 3:** Direct LS‐TA results in patients with viral infection compared to those with Kawasaki disease (KD).

DIRECT LS‐TA	Dataset	GSE73461		GSE68004	
		Multiples of median (MoM) values mean±SD	‐Log_10_(*p* value of *t*‐test)	Multiples of median (MoM) values mean±SD	‐Log_10_(*p* value of *t*‐test)
Log (*IFI27/PSAP*)	KD	0.57 ± 2.14	29.13	0.24 ± 1.83	10
	Viral	5.25 ± 2.20		3.48 ± 1.25	
Log (*IFI27/CTSS*)	KD	0.54 ± 2.16	30.00	0.34 ± 1.85	9.21
	Viral	5.52 ± 2.41		3.80 ± 1.43	
Log (*IFI44L/PSAP*)	KD	0.45 ± 1.98	19.07	0.41 ± 2.04	4.46
	Viral	3.43 ± 1.65		2.27 ± 1.36	
Log (*IFI44L/CTSS*)	KD	0.46 ± 1.97	21.53	0.25 ± 2.08	5.09
	Viral	3.76 ± 1.77		2.35 ± 1.39	
Log (*SIGLEC1/PSAP*)	KD	0.10 ± 0.52	21.49	0.01 ± 0.49	6.58
	Viral	1.20 ± 0.75		0.63 ± 0.34	
Log (*SIGLEC1/CTSS*)	KD	0.05 ± 0.63	20.75	0.00 ± 0.59	5.12
	Viral	1.46 ± 1.02		0.85 ± 0.58	

The conventional IFN score, calculated as the average of five ISGs (*IFIT1, IFITM3, SIGLEC1, IFI27, IFI44L*) normalised to three housekeeping genes (*GAPDH, ACTB, RPL31*), was also significantly elevated in VD patients (*p* < 1e‐9). However, this method required quantification of eight genes compared to only two genes needed for DIRECT LS‐TA measurements.

### 3.2 Comparative Analysis of Diagnostic Performance

**FIGURE 2:**
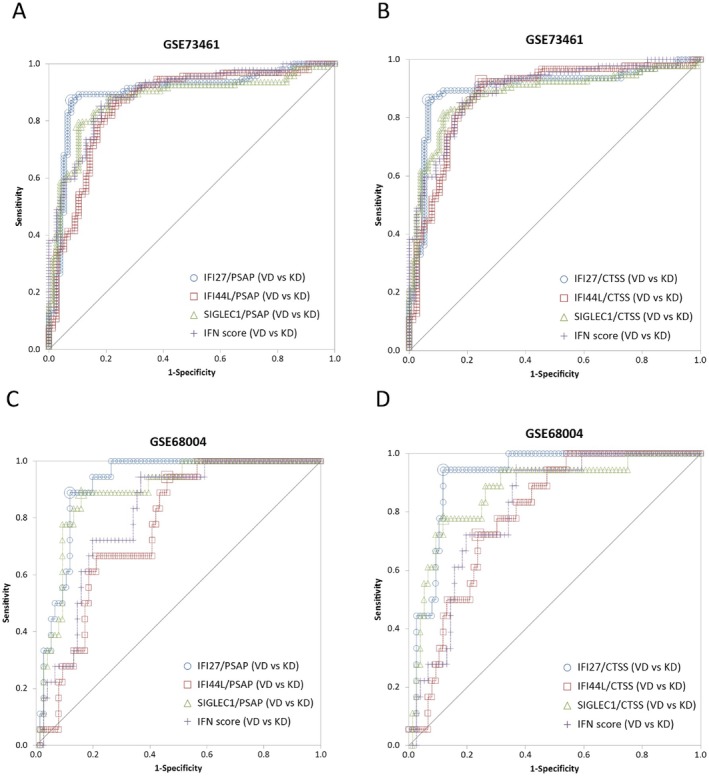
Receiver operating characteristic (ROC) curve of biomarkers in GSE73461 (A and B) and GSE68004 (C and D).

To evaluate the clinical utility of these biomarkers, we performed detailed ROC curve analyses. The results are shown in Table 4 and Figure 2.

**TABLE 4:** Performance metrics of different biomarkers in differentiating Kawasaki disease from viral infection.

Biomarker	AUC, 95% confidence interval		Sensitivity, 95% confidence interval		Specificity, 95% confidence interval	
	GSE73461	GSE68004	GSE73461	GSE68004	GSE73461	GSE68004
DIRECT LS‐TA *IFI27/PSAP*	0.90, 0.85–0.96	0.93, 0.86–0.97	0.87, 0.79–0.93	0.89, 0.65–0.99	0.92, 0.84–0.97	0.88, 0.79–0.94
DIRECT LS‐TA *IFI27/CTSS*	0.90, 0.85–0.95	0.92, 0.86–0.98	0.86, 0.78–0.92	0.94, 0.73–0.99	0.94, 0.85–0.98	0.88, 0.79–0.94
DIRECT LS‐TA *IFI44L/PSAP*	0.86, 0.80–0.92	0.76, 0.66–0.87	0.87, 0.79–0.93	0.94, 0.73–0.99	0.77, 0.66–0.86	0.54, 0.42–0.65
DIRECT LS‐TA *IFI44L/CTSS*	0.88, 0.82–0.93	0.79, 0.69–0.89	0.93, 0.85–0.97	0.72, 0.47–0.90	0.75, 0.64–0.84	0.76, 0.65–0.85
DIRECT LS‐TA *SIGLEC1/PSAP*	0.88, 0.82–0.93	0.89, 0.81–0.96	0.79, 0.69–0.86	0.89, 0.65–0.99	0.90, 0.81–0.96	0.84, 0.74–0.92
DIRECT LS‐TA *SIGLEC1/CTSS*	0.88, 0.83–0.94	0.87, 0.78–0.97	0.81, 0.71–0.88	0.78, 0.52–0.94	0.88, 0.79–0.95	0.88, 0.79–0.94
Conventional IFN score	0.89, 0.85–0.94	0.81, 0.71–0.90	0.85, 0.76–0.92	0.94, 0.73–0.99	0.82, 0.71–0.90	0.63, 0.51–0.74

ROC analysis of *IFI27/PSAP* showed the highest AUC at 0.9, followed by the conventional IFN score at 0.89. VD patients had a significant IFN response, while little activation of IFN‐stimulated genes was seen in KD. This could be useful in the differentiation of the 2 conditions, particularly applied as an exclusion biomarker. That is, if IFN is activated, it is unlikely to be KD; vice versa.

### 3.3 Post‐Test Probability Analysis

Assuming pre‐test odds of 0.1 for KD and the sensitivity and specificity are set to 0.9, if a child is suspected of KD clinically and the child has an elevated *IFI27*, then the post‐test probability of KD is down to 1/82 ~ 1%, which is illustrated in Table 5.

**TABLE 5:** 2X2 table for illustrating the calculation of post‐test probability.

		VD	KD	Total
*IFI27/PSAP*	Elevated	90 × 0.9 = 81	10–9 = 1	82
	Not Elevated	90–81 = 9	10 × 0.9 = 9	18
Total		90	10	

*Note:* Assume specificity and sensitivity of the test are both set to 0.9; a child is suspected of KD clinically; pre‐test odds assumed to be 0.1. If the child has an elevated IFI27, the post‐test probability of KD is down to 1/82 ~ 1%.

## 4 Discussion

As previously reported [27], host response biomarkers play crucial roles in patient triage at emergency departments. For KD, early differentiation from VD is particularly important as prompt treatment with intravenous immunoglobulin can prevent coronary complications. While CRP and procalcitonin are commonly used, these serum protein markers provide only non‐specific information about systemic inflammation, without insight into cell‐type‐specific responses.

Recent advances in transcriptome analysis have led to the identification of DEGs as potential biomarkers. However, as demonstrated in previous studies, these DEG approaches face limitations due to confounding effects from varying cell proportions in blood samples, potentially weakening their diagnostic effectiveness [27]. This is particularly relevant in KD, where significant changes in blood cell populations occur during the acute phase.

Our group recently developed the DIRECT LS‐TA method for determining single‐cell‐type gene expression directly from blood samples [19, 20, 21]. Using this approach in the present study, we showed that the monocyte single‐cell type biomarker *IFI27/PSAP* could be used as a rule‐out test for viral infection in patients with a differential diagnosis of KD and viral infection. The correlation between DIRECT LS‐TA results and expression in isolated monocytes remains robust (R^2^ > 0.9), confirming the method's ability to capture cell‐type‐specific information without separation procedures.

Notably, our results show distinct patterns of monocyte responses between KD and VD. While VD showed significant elevation of ISGs through *IFI27/PSAP, IFI44L/PSAP*, and *SIGLEC1/PSAP* ratios, KD patients demonstrated minimal IFN response. This clear distinction provides a valuable diagnostic tool, particularly given the high specificity (0.92) of *IFI27/PSAP* for viral infection.

However, we observed that KD shares similar monocyte activation patterns with bacterial infection, particularly in *VNN1/PSAP* elevation (data not shown). This finding suggests that while DIRECT LS‐TA effectively rules out viral infection, additional clinical and laboratory parameters are needed to distinguish between KD and bacterial infection. This limitation should be considered when implementing these biomarkers in clinical practice. Although we cannot differentiate patients with bacterial infection at this stage, future development of this new type of single‐cell host‐response biomarker may address this question.

Building upon our previous work [19, 21], the DIRECT LS‐TA approach offers several practical advantages over existing technologies. Unlike computational algorithms developed to deconvolute the cell‐count proportion of each cell type presented in a bulk sample using matrix deconvolution, which require whole‐genome expression data or expensive single‐cell sequencing, the DIRECT LS‐TA method has several additional advantages. First, sample collection requires only a standard blood draw, which is routinely performed in febrile patients. Second, the entire laboratory procedure can be completed within 2–3 h, allowing for rapid clinical decision‐making. Third, the method eliminates the need for complex cell separation procedures, which are both time‐consuming and technically demanding. Fourth, the assay can be performed using standard PCR equipment available in most hospital laboratories, making it accessible for widespread implementation.

The timing of sampling remains important, as we observed in our previous study of COVID infection, IFN responses typically peaked within the first 10 days of VD [27]. It coincides with the range of half‐life of circulating monocytes, which has been reported to be between 1 and 7 days [28]. This temporal pattern, combined with age‐related variations in immune responses, should be considered when interpreting results in paediatric populations. These factors may explain some variations observed in previous studies of KD and viral infection biomarkers.

Several limitations of our study should be acknowledged. The analysis relied on public datasets with varying definitions of KD and viral infection. Additionally, sample sizes were relatively small, and the method cannot identify specific viral pathogens. Future prospective studies with standardised criteria and larger cohorts would help validate these findings.

In conclusion, this study extends our previous work on DIRECT LS‐TA [19, 21] to address a specific clinical challenge in paediatric medicine. The method's ability to differentiate KD from viral infection, combined with its practical advantages—simple blood sampling, rapid processing, and standard equipment requirements—makes it a promising tool for clinical implementation. DIRECT LS‐TA will emerge as a new kind of in vitro diagnostics (IVD) which can convey single‐cell‐type gene expression information from peripheral blood samples. The new kind of IVD and the uniqueness of the information, together with the ease of implementation, will make it very useful in clinics.

## Author Contributions

**Kwong‐Sak Leung:** conceptualization. **Dan Huang:** conceptualization, formal analysis. **Geoffrey Mok:** writing – review and editing. **Tsz‐Ki Kwan:** formal analysis, writing – review and editing, writing – original draft. **Suk‐Ling Ma:** writing – review and editing, writing – original draft. **Assunta C. H. Ho:** writing – review and editing. **Hugh Simon Lam:** writing – review and editing. **Nelson L. S. Tang:** conceptualization, writing – original draft, formal analysis, writing – review and editing, supervision.

## Funding

This work was supported by the Innovation and Technology Commission (PsH/130/24), the HKSTP Incubation Programme, the Research Grants Council of Hong Kong SAR, China (14104625), and the Co‐Funding Mechanism on Joint Laboratories with the CAS (JLFS/M‐403/24).

## Disclosure

The authors have nothing to report.

## Ethics Statement

The authors have nothing to report.

## Consent

The authors have nothing to report.

## Conflicts of Interest

The authors declare the following potential conflicts of interest. Nelson LS Tang is the inventor of the patent “Determination of gene expression levels of a cell type” which has been assigned to The Chinese University of Hong Kong. Kwong‐Sak Leung and Nelson LS Tang are shareholders of Cytomics Ltd. Tsz‐Ki Kwan is an employee of Cytomics Ltd. Cytomics Ltd. holds a licence to use a patent related to the DIRECT LS‐TA assay. Patent application pending (CN116334204A).

## Data Availability

Study data used in this paper are available from Gene Expression Omnibus (GEO), maintained by the US National Institutes of Health.
